# Association of Interleukin 8 and Myocardial Recovery in Patients with ST-Elevation Myocardial Infarction Complicated by Acute Heart Failure

**DOI:** 10.1371/journal.pone.0112359

**Published:** 2014-11-12

**Authors:** Trygve Husebye, Jan Eritsland, Harald Arnesen, Reidar Bjørnerheim, Arild Mangschau, Ingebjørg Seljeflot, Geir Øystein Andersen

**Affiliations:** 1 Department of Cardiology, Oslo University Hospital Ullevål, Oslo, Norway; 2 Center for Clinical Heart Research, Oslo University Hospital Ullevål, Oslo, Norway; 3 Center for Heart Failure Research, University of Oslo, Oslo, Norway; 4 Faculty of Medicine, University of Oslo, Oslo, Norway; Johns Hopkins University SOM, United States of America

## Abstract

**Background:**

No data from controlled trials exists regarding the inflammatory response in patients with de novo heart failure (HF) complicating ST-elevation myocardial infarction (STEMI) and a possible role in the recovery of contractile function. We therefore explored the time course and possible associations between levels of inflammatory markers and recovery of impaired left ventricular function as well as levosimendan treatment in STEMI patients in a substudy of the LEvosimendan in Acute heart Failure following myocardial infarction (LEAF) trial.

**Methods:**

A total of 61 patients developing HF within 48 hours after a primary PCI-treated STEMI were randomised double-blind to a 25 hours infusion of levosimendan or placebo. Levels of IL-6, CRP, sIL-6R, sgp130, MCP-1, IL-8, MMP-9, sICAM-1, sVCAM-1 and TNF-α were measured at inclusion (median 22 h, interquartile range (IQR) 14, 29 after PCI), on day 1, day 2, day 5 and 6 weeks. Improvement in left ventricular function was evaluated as change in wall motion score index (WMSI) by echocardiography.

**Results:**

Only circulating levels of IL-8 at inclusion were associated with change in WMSI from baseline to 6 weeks, r = ÷0.41 (p = 0.002). No association, however, was found between IL-8 and WMSI at inclusion or peak troponin T. Furthermore, there was a significant difference in change in WMSI from inclusion to 6 weeks between patients with IL-8 levels below, compared to above median value, ÷0.44 (IQR÷0.57, ÷0.19) vs. ÷0.07 (IQR÷0.27, 0.07), respectively (p<0.0001). Levosimendan did not affect the levels of inflammary markers compared to control.

**Conclusion:**

High levels of IL-8 in STEMI patients complicated with HF were associated with less improvement in left ventricular function during the first 6 weeks after PCI, suggesting a possible role of IL-8 in the reperfusion-related injury of post-ischemic myocardium. Further studies are needed to confirm this hypothesis.

**Trial Registration:**

ClinicalTrials.gov NCT00324766

## Introduction

Inflammation is thought to play an important role in the pathophysiology of heart failure (HF). Several human studies in patients with chronic HF have shown high levels of circulating cytokines and a clear association with severity (NYHA class) and prognosis of the disease [1], [2], [3]. The role of inflammation in acute HF syndromes is less clear, especially in patients with HF complicating ST-elevation myocardial infarction (STEMI). De novo HF in STEMI patients is associated with large myocardial infarctions (MI) and persistent inflammation has been proposed to play a role in infarct expansion and adverse ventricular remodelling that may contribute to the poor prognosis in these patients [4]. However, data are lacking from clinical trials on the inflammatory response both regarding the time-course and possible associations to left ventricular function in patients with de novo HF after STEMI. A possible association between inflammation and recovery of myocardial function after a primary percutaneous coronary intervention (PCI) treated STEMI is also unknown.

The LEAF (LEvosimendan in Acute heart Failure following myocardial infarction) trial was a randomized, placebo controlled study in patients with de novo HF following a PCI-treated STEMI [5]. The endpoint of this study was improvement in contractility in post-ischemic myocardium, measured as change in wall motion score index (WMSI). Treatment with levosimendan in patients with decompensated HF has been shown to reduce levels of pro-inflammatory cytokines [6], [7]. Possible anti-inflammatory effects of levosimendan in de novo HF following acute mycordial infarction (AMI) has so far not been adressed in clinical trials.

The aims of the present substudy were to explore the time-course of the inflammatory response pattern and study possible associations between inflammation and impaired left ventricular (LV) function and myocardial injury. A secondary aim was to investigate wether levosimendan would influence levels of inflammatory markers in patients with acute HF following PCI-treated STEMI.

## Materials and Methods

The protocol for this trial and supporting CONSORT checklist are available as supporting information; se Checklist S1 and Protocol S1. The Regional Ethics Committee South–Eastern Norway Regional Health Authority approved the study October 11, 2004 (reference 538-04218), which was conducted in accordance with the principles of the Declaration of Helsinki and all patients provided written informed consent. The study was registered at www.clinicaltrial.gov; identifier: NCT00324766.

The LEAF trial was an investigator initiated, manufacturer independent study conducted at Oslo University Hospital, Ullevål in Oslo, Norway. Patients were included between April 20, 2006 and December 13, 2010 with follow-up completed May 3, 2011. Due to technical reasons (a delay in the registration process with Oslo University Hospital as sponsor) 2 patients were included (April 20, April 25) before the trial was registered at the clinicaltrial.gov (May 10). The authors confirm that all ongoing and related trials for this drug/intervention are registered at clinicaltrials.gov.

### Study design and population

The LEAF trial was a randomized, double blind, placebo-controlled, single-centre, parallel-group study. Details on study design and main results have recently been published [5]. Briefly, patients were assigned to a 25 h infusion (0.2 µg/kg/min for one h followed by 0.1 µg/kg/min for 24 h) of levosimendan or placebo. Included in the study were patients with acute HF (including patients in cardiogenic shock) complicating a primary PCI treated STEMI. The inclusion criteria were: (1) opening of an occluded or dilation of a stenotic coronary artery presumed to be the infarct related artery, (2) signs of decreased wall-motion in at least three of 16 segments of the left ventricle with echocardiography, (3) clinical HF defined as dyspnoea at rest at screening and at least one of the following signs within 48 h after PCI: pulmonary oedema, signs of marked pulmonary congestion on chest x-ray, need for continuous positive airway pressure or mechanical ventilation, and need for i.v. diuretics due to symptoms of congestion or persistent oliguria (urine output <0.5 mL/kg/h) after volume therapy. Additional criteria in the cardiogenic shock subgroup: (1) systolic blood pressure (SBP) <90 mmHg after 60 min of adequate volume therapy or SBP between 90 and 100 mmHg in spite of inotropic support by catecholamine infusion and (2) signs of organ hypo-perfusion such as oliguria, cold and clammy extremities or reduced consciousness.

The exclusion criteria were: age<20 years, heart rate>120 beats per minute, septic shock, acute respiratory distress syndrome, creatinine>450 µmol/l, severe hepatic failure, significant mechanical outflow obstruction, allergy against study medication or one of its components, anaemia (haemoglobin <8 g/dl) or pregnancy.

### Laboratory methods

Blood samples were drawn at the time of inclusion, after 25 h, 2 days, 5 days and 6 weeks for routine analyses and for determination of interleukin-6 (IL-6), C-reactive protein (CRP), soluble interleukin-6 receptor (sIL-6R), soluble glycoprotein 130 (sgp130), monocyte chemoattractant protein-1 (MCP-1), interleukin-8 (IL-8), matrix metalloproteinase-9 (MMP-9), soluble vascular cell adhesion molecule-1 (sVCAM-1), soluble intercellular adhesion molecule-1 (sICAM-1) and tumor necrosis factor-α (TNF-α). Serum was separated within 1 hour by centrifugation at 2500 g for 10 min and used for all analyses, except MCP-1, which was determined in citrated plasma, stored on ice and separated within 30 min by centrifugation at 4°C and 3000 g for 20 min to obtain platelet-poor plasma. All blood samples were stored at ÷80°C until analyzed.

CRP was determined by enzyme-linked immunosorbent assays (DRG instruments, Marburg/Lahn, Germany). Il-6, sIL6-R, sgp130, MCP-1, IL-8, MMP-9, sVCAM-1, sICAM-1 and TNF-α were all measured by enzyme immunoassays from R & D Systems Europe (Abingdon, Oxford, UK). In our laboratory, the inter-assay coefficients of variation were as follows, IL-6 10.5%, CRP<5%, sIL-6R 3.6%, sgp130 5.2%, MCP-1 9.2%, IL-8 7.6%, MMP-9 7.3%, sICAM-1 6.6%, sVCAM-1 5.3% and TNF-α 8.5%.

### Echocardiography

LV function was measured as WMSI by echocardiography in accordance with current guidelines [8]. A 16-segment model was used where a normally or hyperkinetic contracting segment was assigned a score of 1, hypokinetic 2, akinetic 3 or dyskinetic 4. The WMSI was calculated as the sum of scores divided by the number of segments scored. Echocardiography was performed at inclusion, after 25 hours, 5 days and 6 weeks. Two experienced echo-cardiographers performed all examinations, and a single observer performed all analyses. Examinations were performed with a digital ultrasonic device system (Vivid i or Vivid 7, GE Vingmed Ultrasound, Horten, Norway) and analysed with dedicated software (Echopac, GE Vingmed Ultrasound).

### Measurement of infarct size

Perfusion imaging was performed after 6 weeks by 99 m-tetrofosmin ECG-gated myocardial single photon emission computed tomography (SPECT). Infarct size (proportion perfusion defect) was expressed as percentage of LV mass [9].

### Statistical analyses

Analyses of continuous data that were normally distributed were performed by two-sample t-tests. Otherwise, non-parametric tests were used throughout. Differences between groups were tested by Mann-Whitney U-test, Wilcoxon signed rank test was used to assess within-group changes from baseline to day 1, day 2, day 5 and 6 weeks only when Friedman’s test showed statistically significant changes. Correlation analyses were performed using the Spearman rho. A significance level of 5% with a two-tailed test was used. Statistical analyses were performed using IBM SPSS version 18.0 (SPSS Inc., Chicago, IL, USA).

## Results

### Patient population

**Figure 1:**
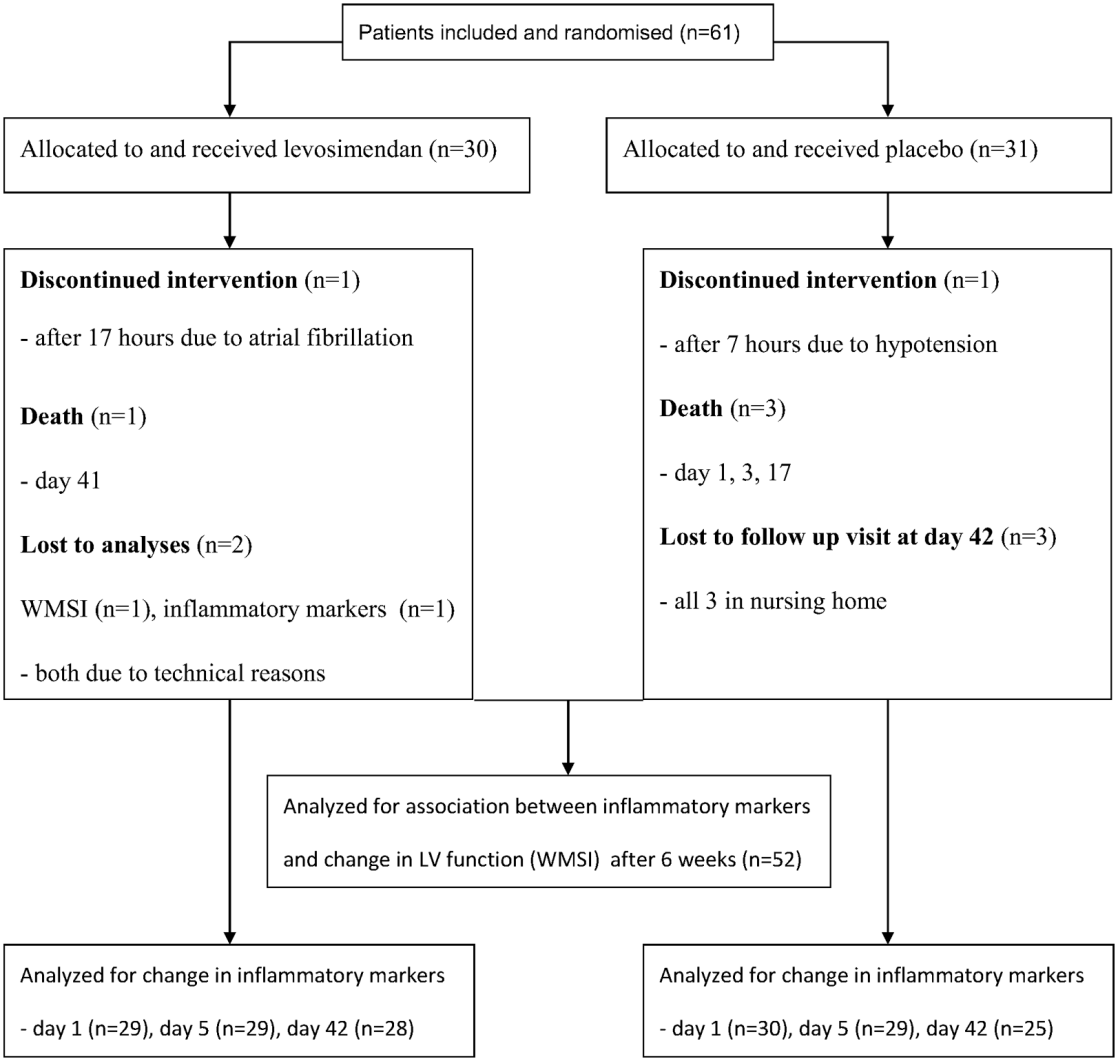
Study-flow diagram of included patients.

A total of 61 patients were included in the LEAF trial. Flow chart and characteristics of the patients analyzed in the present study are shown in Figure 1 and Table 1.

**Table 1:** Patient characteristics at inclusion.

	Levosimendan (n = 30)	Placebo (n = 31)	Analyzed at 6 week follow-up (n = 52)
Age	66 (56, 74)	62 (56, 74)	64 (56, 74)
Female, n (%)	12 (40)	6 (19)	17 (33)
Hypertension, n (%)	10 (33)	11 (36)	17 (33)
Dyslipidemia, n (%)	3 (10)	10 (32)	9 (17)
Diabetes mellitus, n (%)	5 (17)	1 (3)	6 (12)
Current smoking, n (%)	12 (41)	10 (33)	18 (35)
Previous MI, n (%)	7 (23)	4 (13)	9 (17)
Multivessel disease, n (%)	13 (43)	18 (58)	25 (48)
Cardiogenic shock, n (%)	4 (13)	5 (16)	6 (12)
IABP at baseline, n (%)	8 (27)	9 (29)	11 (21)
Hours from symptoms to PCI	3 (2–8)	3 (2, 6)	3 (2, 6)
Hours from PCI to baseline	24 (14, 33)	22 (14, 26)	22 (14, 32)
TnT peak, ng/L	12195 (7990, 16187)	11828 (5670, 18640)	12915 (7990, 16607)
Creatinine, µmol/L	82 (66, 90)	82 (73, 110)	81 (67, 91)
Systolic BP, mmHg	102 (93, 114)	107 (93, 115)	104 (94, 115)
NT-proBNP (pmol/L)	386 (297, 597)	474 (248, 922)	422 (261, 784)
WMSI at baseline	1.94 (1.75, 2.13)	2.0 (1.88, 2.19)	1.94 (1.81, 2.13)
LVEF at baseline, %	43 (38, 49)	40 (33, 47)	43 (38, 48)

Continuous data are presented as median (IQR) unless indicated otherwise. MI, myocardial infarction; IABP, intra-aortic balloon counter-pulsation; PCI, percutaneous coronary intervention; TnT, troponin T; BP, blood pressure; NT-proBNP, N-terminal pro B-type natriuretic peptide; WMSI, wall motion score index; LVEF, left ventricular ejection fraction.

### Inflammatory markers

Blood samples were drawn at the time of inclusion 22 h (median value, interquartile range (IQR) 14, 29) after PCI.

#### Association between inflammatory markers and improvement in left ventricular function

**Figure 2:**
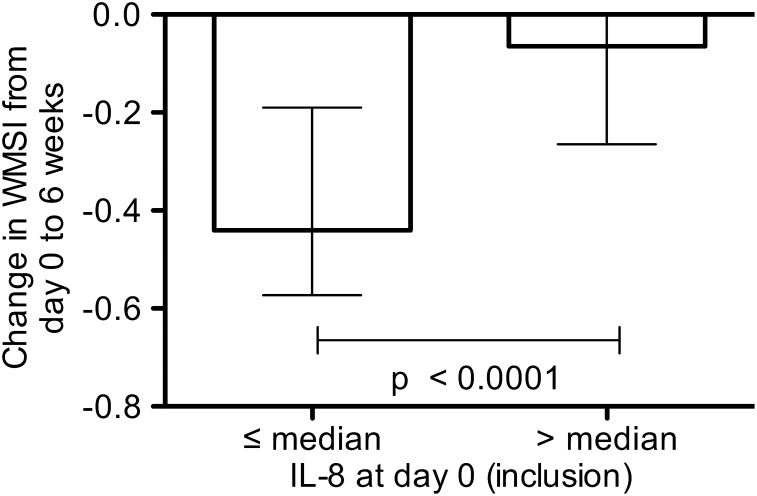
Changes in left ventricular function according to levels of IL-8 in patients with ST-elevation myocardial infarction developing symptomatic heart failure. Changes in wall motion score index from inclusion (median 22 h, interquartile range 14, 29 after PCI) to 6 weeks. Patients (n = 52) were dichotomized into groups according to a level of IL-8 at inclusion below (10.0−≤25.6 pg/mL) or above (25.7–209 pg/mL) median value. Data are presented as median values with interquartile range.

LV function measured as WMSI improved during the 6 weeks follow up period. WMSI was 1.63 (IQR 1.50, 1.93) at 6 weeks compared to 1.94 (IQR 1.81, 2.13) at inclusion. Of the inflammatory markers measured, only IL-8 was associated with improvement in LV function over time, measured as change in WMSI. Circulating levels of IL-8 measured at inclusion were correlated with WMSI after 6 weeks and change in WMSI from inclusion to 6 weeks, r = 0.42 (p = 0.002) and r = ÷0.41 (p = 0.002), respectively. IL-8 levels at inclusion, however, were not correlated with WMSI measured at the same time point, r = 0.16 (p = 0.24), peak troponin T (TnT), r = 0.21 (p = 0.11) or infarct size at 6 weeks, r = 0.15 (p = 0.31). There was a significant between-group difference in change in WMSI from inclusion to 6 weeks between patients with low (≤ median value) compared to high IL-8 levels (> median value) at inclusion,÷0.44 (IQR÷0.57, ÷0.19) vs. ÷0.07 (IQR÷0.27, 0.07) (p<0.0001) (Figure 2). This difference in improvement in WMSI according to levels of IL-8 at inclusion was present already after 5 days (p = 0.0004). Furthermore, patients with worsening or no improvement in WMSI 6 weeks after the STEMI (n = 12) had significantly higher IL-8 levels at inclusion compared to patients with improved WMSI during the post-infarction period (n = 40), 31.2 pg/mL (IQR 28.2, 66.7) vs. 22.8 pg/mL (IQR 17.1, 29.9) (p = 0.002). Patient characteristics at inclusion, stratified to levels of IL-8 ≤ or > median are shown in Table 2.

**Table 2:** Patient characteristics at inclusion, stratified to levels of IL-8 ≤ or > median value.

	IL8≤25.6 pg/mL (n = 26)	IL8>25.6 pg/mL (n = 26)	p value
Age, years	66 (53, 74)	62 (56, 73)	0.69
Female, n (%)	8 (31)	8 (31)	1.0
Hypertension, n (%)	5 (19)	12 (46)	0.08
Dyslipidemia, n (%)	5 (19)	4 (15)	1.0
Diabetes mellitus, n (%)	3 (12)	3 (12)	1.0
Current smoking, n (%)	8 (31)	11 (42)	0.57
Previous MI, n (%)	3 (12)	6 (23)	0.47
Multivessel disease, n (%)	12 (46)	13 (50)	1.0
Cardiogenic shock, n (%)	1 (4)	5 (19)	0.19
IABP at baseline, n (%)	3 (12)	8 (31)	0.17
Hours from symptoms to PCI	3 (2, 5)	4 (2, 7)	0.25
Hours from PCI to baseline	26 (16, 37)	19 (12, 23)	0.04
TnT peak, ng/L	11211 (6098, 15925)	13570 (9284, 18025)	0.15
Creatinine, µmol/L	82 (73, 91)	79 (60, 98)	0.52
Systolic BP, mmHg	110 (99, 121)	99 (92, 110)	0.02
CRP (mg/L)	53 (38, 114)	60 (32, 80)	0.49
NT-proBNP (pmol/L)	422 (272, 695)	361 (256, 735)	0.95
WMSI	1.93 (1.75, 2.06)	1.94 (1.81, 2.19)	0.41
LVEF %	43 (39, 49)	43 (35, 48)	0.34
Medication before hospitalization			
Statins	5 (19)	6 (24)	0.74
Aspirin	4 (159	9 (36)	0.12
ACE-inh/ARB	4 (15)	7 (28)	0.32

Continuous data are presented as median (IQR) unless indicated otherwise. MI, myocardial infarction; IABP, intra-aortic balloon counter-pulsation; TnT, troponin T; BP, blood pressure; CRP, C-reactive protein, NT-proBNP, N-terminal pro B-type natriuretic peptide; WMSI, wall motion score index; LVEF, left ventricular ejection fraction; ACE-inh, angiotensin-converting enzyme inhibitor; ARB, angiotensin-receptor blocker.

#### Association between levels of inflammatory markers and infarct size after 6 weeks

Infarct size determined by SPECT after 6 weeks (n = 48) was 45% (IQR 36, 51) of LV mass. A moderate negative correlation was found between infarct size and levels of sIL-6R on days 1 and 5, r = ÷0.32 (p = 0.035) and r = ÷0.38 (p = 0.007), respectively, otherwise no associations between the different markers and infarct size were found.

#### Changes over time compared to levels at inclusion

**Figure 3:**
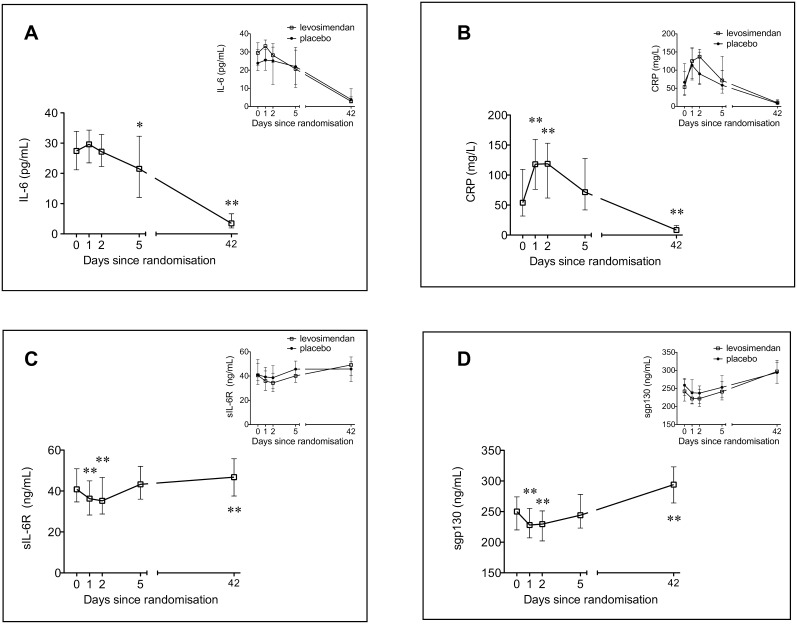
Temporal profiles of circulating inflammatory markers in patients with ST-elevation myocardial infarction developing symptomatic heart failure. Levels of IL-6 (A), CRP (B), sIL-6R (C) and sgp130 (D), during the first 6 weeks after inclusion (median 22 h, interquartile range 14, 29, after PCI), (n = 61). Inset: The results according to randomization groups, levosimendan (n = 30) or placebo (n = 31). Median values with interquartile range. * Change from baseline, p<0.05 ** Change from baseline, p<0.001.

**Figure 4:**
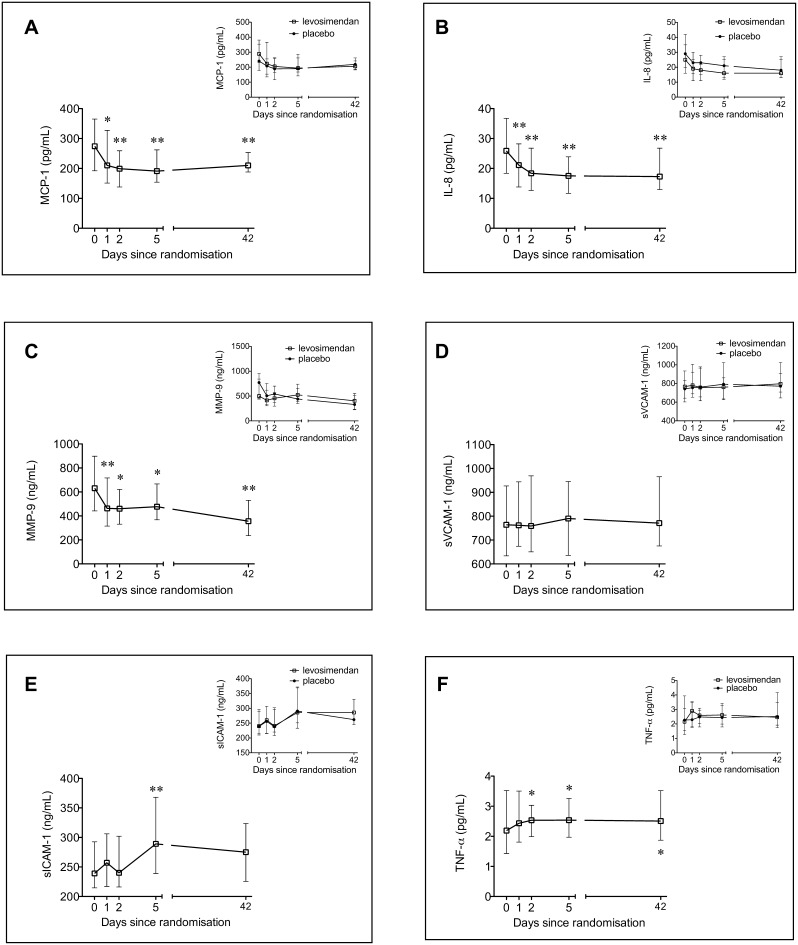
Temporal profiles of circulating inflammatory markers in patients with ST-elevation myocardial infarction developing symptomatic heart failure. Levels of MCP-1 (A), IL-8 (B), MMP-9 (C), sVCAM-1 (D), sICAM-1 (E) and TNF-α (F) during the first 6 weeks after inclusion (median time 22 h, interquartile range 14, 29, after PCI), (n = 61). Inset: The results according to randomization groups, levosimendan (n = 30) or placebo (n = 31). Median values with interquartile range. * Change from baseline, p<0.05 ** Change from baseline, p<0.001.

The time courses of the measured inflammatory markers are shown in Figure 3 and 4. IL-6 and CRP increased during the acute STEMI while the levels of sIL-6R and sgp130 were lower compared to measurements at stable conditions 6 weeks later (Figure 3). MCP-1, IL-8 and MMP-9 levels were highest at baseline compared to all other time points (Figure 4). TNF-α levels increased modestly from inclusion to day 2 and remained unchanged in the 6 weeks study period (Figure 4).

#### Effect of levosimendan treatment on markers of inflammation

There were no statistically significant between-group differences in changes from randomization (inclusion) during the study period of IL-6, CRP, sIL-6R, sgp130, MCP-1, IL-8, MMP-9, sVCAM-1, sICAM-1 and TNF-α, at the different time points (Figure 3 and 4, insets).

## Discussion

The present substudy of the LEAF trial is the first to explore possible associations between inflammatory markers and LV function and myocardial injury in patients developing acute HF following PCI-treated STEMI. The main finding of the study was that circulating levels of IL-8, measured at the time of inclusion (median 22 h after PCI), were highly associated with improvement in LV function during the first 6 weeks after STEMI. High levels of IL-8 have recently been shown to be associated with adverse clinical outcome in patients with chronic HF [10]. We found that high levels of IL-8 were associated with less improvement in LV function during the first 6 weeks after STEMI. Interestingly, this association between IL-8 and recovery of LV function was present without any association to peak TnT or final infarct size after 6 weeks suggesting that IL-8 levels may reflect other aspects of myocardial injury than necrosis. IL-8 plays a fundamental role in regulating neutrophil infiltration in ischemic and reperfused myocardium [11]. Activated neutrophils release several factors which can cause tissue and endothelial damage. Additionally, neutrophils can form aggregates that plug capillaries, contributing to microvascular obstruction (MVO) [12]. MVO was in an experimental animal model of reperfused infarcted myocardium found to be associated with adverse LV remodelling and segmental function, beyond and independent of infarct size [13]. MVO has been shown to be associated with reduced myocardial salvage after primary PCI in STEMI patients, and MVO was a stronger predictor of LV remodelling than peak creatine kinase and infarct size [14], [15], [16]. It therefore seems that MVO may be associated with LV remodelling and impaired function also by mechanisms not related to myocardial necrosis and infarct size alone. Hypothetically, IL-8 may, by its effects on neutrophils, be part of an inflammatory cascade that contributes to such injury of the reperfused myocardium. Although there were trends in differences in baseline characteristics between patients with high vs. low Il-8 levels, the data suggest that differences in IL-8 levels probably are more related to the acute MI and the accompanying degree of heart failure than the patients’ medical history before the index MI. Although mainly non-significant trends, due to the relatively low number of patients, more patients in the group with high levels of IL-8 were in cardiogenic shock or had an IABP inserted, and patients with high IL-8 levels had higher peak TnT levels and lower systolic blood pressure (Table 2).

The time courses of different markers like IL-6, CRP and TNF-α were consistent with previous studies on patients with either acute decompensated HF or acute MI [17], [18], [19]. Our data, however, is from a STEMI population with very large MIs and severe HF, including cardiogenic shock. Median infarct size was 45% of LV mass, while infarct size in STEMI patients reported from our institution in the same time period was 14% when unstable patients with HF were excluded [20]. Patients with severe HF and especially cardiogenic shock are usually excluded from MI studies and our data on the circulating levels and temporal profile of previously described as well as novel inflammatory markers provide new knowledge [21].

Our data indicate that the initial powerful rise in IL-6 coincides with a consumption of both sIL-6R and sgp130. A decline in sIL6-R, as shown in our study, has previously been reported in a few, smaller studies in patients with acute MI [22], [23]. Recently, in a cross-sectional cohort study of 1028 STEMI patients we found no association between levels of IL-6, sgp130 and sIL-6R, measured median 18 hours after PCI, and myocardial injury measured as peak TnT [24]. Our data on STEMI patients with acute de novo HF are in accordance with these results.

We have previously shown that levosimendan improved LV function compared to placebo in post-ischemic myocardium in patients developing acute HF following a PCI-treated STEMI, however no significant effect on levels of N-terminal proB-type natriuretic peptide (NT-proBNP) was found [5]. The lack of significant between-group differences in changes of the measured markers during the study period between levosimendan and placebo differ from findings in previous studies on patients with HF [6], [7], [25], [26]. However, patients in these studies had decompensated chronic HF with LV ejection fraction <35% and acute MI was an exclusion criterion. Our patients had rather low levels of NT-proBNP, consistent with absence of longstanding pressure or volume overload, factors that are believed to be important in the inflammatory response seen in patients with both chronic and acute decompensated HF [27]. The myocardial injury related to the PCI-treated STEMI was in our patients probably a more powerful stimulus to the inflammatory response than the acute development of HF [11], [28].

### Limitations

Due to the study design, the first blood sampling was performed median 22 hours after PCI. We were therefore not able to evaluate the levels of circulating inflammatory markers during the first hours after opening of the infarct related artery and may have missed the peak level of the different markers in relation to the STEMI. However, as the aim of the LEAF trial was to investigate the effect of levosimendan on STEMI patients with symptomatic HF, we could not include patients immediately after PCI, as many of the patients developed HF several hours after PCI.

The LEAF trial was not designed to evaluate possible associations between specific inflammatory markers and LV function or infarct size. The number of participants may have been too low to detect other possible associations between levels of inflammatory markers and impaired LV function. Possible association between inflammatory mediators and levosimendan treatment was a prespecified endpoint, but the analyses regarding LV function and infarct size were performed on a post-hoc basis and must be interpreted with caution.

### Conclusion

High levels of IL-8 in STEMI patients complicated with HF were associated with less improvement in LV function during the first 6 weeks after PCI, suggesting a possible role of IL-8 in the reperfusion-related injury and adverse LV remodelling of post-ischemic myocardium. Further studies are needed to confirm this hypothesis.

## Supporting Information

Checklist S1 **CONSORT Checklist S1.**
(DOC)

Protocol S1 **The full version of the trial protocol: LEAF Studyprotocol S1.**
(DOC)

Protocol S2 **LEAF Studyprotocol First Amendment S2.**
(DOC)

Protocol S3 **LEAF Studyprotocol Second Amendment S3.**
(DOC)

Data Availability S1 **Supporting information File S1.**
(XLS)
