# Supplementary material for: Association of Interleukin 8 and Myocardial Recovery in Patients with ST-Elevation Myocardial Infarction Complicated by Acute Heart Failure
Source: PLoS One. 2014 Nov 12;9(11):e112359. doi: 10.1371/journal.pone.0112359 (PMC4229310; doi:10.1371/journal.pone.0112359)
Supplement: Protocol S1 — The full version of the trial protocol: LEAF Studyprotocol S1. (DOC) [file pone.0112359.s002.doc]

# CLINICAL STUDY PROTOCOL

Title: Safety and efficacy of levosimendan in patients with acute myocardial infarction complicated by symptomatic left ventricular failure

Sponsors protocol nr: 0105

Vs. 02

Revised: 2004-01

Registered at : www.clinicaltrials.gov

**Phase** III study.

**Design:** Double blind, placebo-controlled study with parallel groups in patients undergoing acute percutaneous-coronary intervention (PCI) for acute myocardial infarction complicated with decompensated, symptomatic heart failure. The study includes a prospectively defined subgroup of patients in cardiogenic shock.

**Purpose:**

Evaluate the safety and efficacy of a 24-hour infusion with levosimendan compared to placebo, in patients with acute myocardial infarction complicated with decompensated heart failure after acute revascularization by PCI.

**Responsible for the study**: Department of Cardiology, Ullevaal University hospital

**Principle investigator:** Dr Geir Øystein Andersen (MD, PhD)

## PROTOCOL SUMMARY

| Phase | III |
| --- | --- |
| **Design** | Double blind, placebo-controlled study with parallel groups in patients with acute PCI treated myocardial infarction complicated with decompensated heart failure. Prospectively defined subgroup of patients in cardiogenic shock. |
| **Purpose** | Evaluate the safety and efficacy of a 24-hour infusion with levosimendan compared to placebo, in patients with acute myocardial infarction complicated with decompensated heart failure and in a subgroup of patients in cardiogenic shock. |
| **Primary endpoint** | **Efficacy parameters**:   - - Changes from baseline to 5 days in Wall motion score-index measured by echocardiography.   - Changes from baseline to 5 days in BNP (Brain natriuretic peptide).   - Clinical composite at 5 days after start of the study drug infusion, according to the physician’s global assessment measured as change from baseline to 5 days.   **Safety parameters**: Number of patients developing:   - - Hypotension: BP < 90 mm Hg or a drop in mean arterial BP > 10 mm Hg in patients with cardiogenic shock.   - Tachycardia (heart rate above 120).   - Atrial fibrillation.   - Ventricular arrhythmia (VT, VF, TDP).   - Ischemic episodes (ECG changes or chest pain demanding treatment). |
| **Secondary endpoints** | Efficacy parameters  - MACE: Time to death, non-fatal myocardial infarction or revascularization of the infarct related artery during the first 6 weeks and 6 month after start of the study drug infusion. - Changes from baseline to 24 hours in Wall motion score-index measured by echocardiography - Changes from baseline to 6 weeks in Wall motion score-index measured by echocardiography - Changes from baseline to 24 hours in BNP - Changes from baseline to 6 weeks in BNP - Number of days in intensive or coronary care unit. - Total number of days hospitalised. - Time to re-hospitalization for decompensated heart failure. - Clinical composite at 24 hours after the start of the study drug infusion, according to the physician’s global assessment measured as change from baseline to 24 hours. - Infarct size measured at 6 weeks by gated spect (radioisotope technique). - Changes of inflammation markers, haemostasis parameters and serum lactate from baseline to 24 hours, 5 days and 6 weeks. - Changes of E/E` ratio measured by echocardiography from baseline to 24 hours and 5 days.   **In a subgroup of patient *with* cardiogenic shock:**   - Improvement of hemodynamic parameters (CI, PCWP, and SVRI) from baseline to 24 and 48 hours. - Number of days on intra-aortic-balloon counter pulsation treatment (IABP). - Improvement of central venous oxygen saturation. - Improvement of kidney function measured as creatinine clearance. - Total mortality (number of patients alive at discharge).   **In patients *without* cardiogenic shock:**   - - The patient’s global assessment VAS-scale at 24 hours and at 5 days after the start of the study drug infusion.   - Need for IV inotropes, at the clinicians decision, after start of the study drug infusion. |
| **Number of patients** | 60 patients at 1 centre.  A minimum of 14 patients in cardiogenic shock will be included. |
| **Patient group**  **Major inclusion criteria:** | Patients who are hospitalised with acute ST-segment elevation myocardial infarction subject to acute PCI  *Or*  Patients with non-ST segment elevation myocardial infarction subject to PCI within 72 hours after start of chest pain.  *and all of the following (1-3)*   - - Revascularization by PCI with opening of an occluded coronary artery or balloon dilatation of a stenotic coronary artery presumed to be culprit lesion.   - Depressed contractility of the left ventricle (left ventricle ejection fraction < 40 %) assessed by echocardiography   - Dyspnoea at rest at screening and *at least* one of the following signs of left ventricular failure (within 48 hours after PCI):     - Pulmonary edema     - Signs of marked pulmonary congestion on chest x-ray     - Need for CPAP or mechanical ventilation     - Need for IV diuretics.     - Oliguria (<0.5 ml/kg/hour) as a sign of hypoperfusion of the kidneys (after volume therapy) |
| **Subgroup of patients with cardiogenic shock:**  **Additional inclusion criteria** | Additional inclusion criteria includes *both* of the following:   - - Systolic BP <90 mmHg after 60 min of adequate volume therapy or systolic BP between 90 and 100 mmHg with inotropic support by catecholamine infusion   - Signs of hypoperfusion (low-output heart failure)     - Oliguria (diuresis <0.5 ml/kg/hour)     - Cold, clammy extremities     - Reduced consciousness |
| **Exclusion criteria** | - Age below 20 years, - Heart rate above 120 bpm - Septic shock - ARDS - Creatinine >450 μmol/l - Hepatic impairment - Significant mechanical outlet obstruction - Allergy against study drug medication or one of its ingredients - Anaemia (Hb < 8 g/dl) - Pregnancy |
| **Methods** | - Regional contractility or wall motion score-index is measured by echocardiography including tissue doppler imaging at baseline and 24 hours, 5 days and 6 weeks after the start of the study drug infusion.   - Infarct size measured at 6 weeks by gated spect (radioisotope teqniche). - A blood sample is frozen at baseline and after 1, 2, and 5 days and after 6 weeks after the start of the study drug infusion. BNP, lactate and inflammation markers are analysed by methods specified in the study protocol. - Hemodynamic data (Cardiac index, stroke volume, PCWP, SVRI etc.) and central venous oxygen saturation are collected by a pulmonary artery-catheter (Swan-Ganz catheter) in a subgroup of patients in cardiogenic shock. - IV inotropes are allowed to be used freely in the subgroup of patients in cardiogenic shock, but not in the other patients. If IV inotropes is needed, the patient should be included in the cardiogenic shock subgroup. If IV inotropes is needed at the clinician’s decision after the start of the study drug infusion, this should be regarded as an endpoint. - Global assessment is completed by the physician and the patient as change from baseline at 24 hours and at 5 days after the start of study drug treatment. - ”Hospitalisation” is measured according to definitions in the study protocol and mortality and angiographic data is collected. - Safety variables (adverse events, HR, BP, ECG, lab, concomitant medication) are recorded during the study. - A prospectively defined subgroup of patients in cardiogenic shock will be randomised by stratified randomisation. |
| **Study medication** | Bolus dose of 12 ug/kg for 60 minutes. The bolus dose is followed by a continuous infusion of 0,1 ug/kg/min for 24 hours. Noradrenalin must be mixed and ready for use before starting the study drug infusion. If the dose is not well tolerated (systolic BP <80 mm Hg and a drop in MAP >10 mm Hg):  Reduce the dose to 0,05 ug/kg/min. If this is not sufficient:  Start noradrenalin infusion (start dose: 0.03 μg/kg/min). If this is not sufficient: Stop the infusion of the study drug.  After 24 hours the infusion is turned off abruptly.  Placebo is administered in the same way as levosimendan. |
| **Total duration of study** | The study drug infusion is 25 hours long.  There are follow-up during the first 5 days and then at 6 weeks after the start of study drug treatment. The MACE end-point and total mortality will be measured at 6 weeks and at 6 months. |
| **Centres** | Ullevaal University hospital as single centre. |
| **Principle investigator** | Dr Geir Øystein Andersen (MD, PhD) |
| **Steering committee** | Reidar Bjørnerheim (MD, PhD, Head of the Department of Cardiology), Arild Mangschau (MD, PhD, Head of the Coronary care unit), Jan Eritsland (MD, PhD), and Geir Øystein Andersen (MD, PhD). (Department of Cardiology, Ullevaal University hospital, N-0407 Oslo) |
| **Country** | Norway |
| **Draft time plan** | The inclusion period will be starting in 2006.  Inclusion period is estimated to end in 2009.  Results to be available in 2010. |

**Bacground and study rationale:**

Acute myocardial infarction is the result of a partially or fully occluded coronary artery. This results in ischemic myocardium with cell necrosis and scar tissue formation. Reperfusion therapy by thrombolysis or PCI is the key factor in modern treatment of acute myocardial infarction.

The calcium sensitizer levosimendan has been shown to increase hemodynamics and survival in patient hospitalised for decompensated heart failure (1, 2). There are limited data in patients with acute myocardial infarction. Only one major study, the RUSSLAN trial, has reported results on levosimendan in this clinical setting (3). The RUSSLAN trial was primarily a safety and dose-response trial. The incidence of ischemia and/or hypotension was similar in all treatment groups except in the highest levosimendan dose group. Levosimendan-treated patients also showed improved survival at 6 months. In the RUSSLAN trial, acute revascularization and cardiogenic shock were exclusion criteria. Thus, the effects of levosimendan in patients with myocardial infarction treated with modern reperfusion therapy are lacking.

As is the usual case for patient in cardiogenic shock, this group of patients has been excluded from all randomized trials using levosimendan. Evidence of the safety and efficacy of levosimendan in patients with cardiogenic shock is therefore also lacking even though this patient group with critically reduced left ventricular systolic function, should theoretically benefit the most.

**Hypothesis:**

Reperfusion therapy by thrombolysis or acute percutan coronary intervention (PCI) is emerging as routine treatment of acute myocardial infarction. After reperfusion of the ischemic myocardium decreased contractility is seen for several days despite of restoration of epicardial blood flow. This phenomenon is called myocardial stunning (4). The mechanisms underlying this phenomenon are partially unknown, but seem to include increased calcium overload in the myocytes during reperfusion and the production of free oxygen radicals (4). The sum of these effects leads to decreased contractility as a result of decreased calcium sensitivity.

Hypothesis: Treating patients in this critical clinical setting with the calcium sensitizer levosimendan (5) during the first hours after acute PCI will possibly improve contractility without harmful side effects. In the setting of an acute myocardial infarction with signs of decompensated heart failure, levosimendan could improve myocardial function during the stunning period, without increased oxygen consumption and risk of lethal arrhythmias. This could potentially improve patients symptoms, reduce the need for potentially harmful inotropes (6) and decrease the combined endpoint of death, new myocardial infarction and new revascularization during the first 6 month of follow-up. According to this hypothesis, levosimendan will also be able to improve markers of myocardial function and reduce markers of inflammation. In the setting of cardiogenic shock, levosimendan will be safe to use in combination with traditional inotropes. It will improve regional contractility and left ventricular function measured by echocardiography and BNP levels. It will also improve hemodynamics including tissue perfusion, measured by pulmonary artery catheterization.

**Scientific value-summarized**

The present study will provide new knowledge about the effects and safety of levosimendan in the clinical setting of acute myocardial infarction complicated by left ventricular failure in spite of modern rreperfusion therapy by acute PCI.

The study will provide new information about whether increasing the calcium sensitivity of the myofilaments and vasodilatation could improve regional contractility during the stunning period after restoriation of epicardial blood flow by PCI. The double blind, placebo controlled design makes it possible to study the effect of the active study drug on regional contractility during a time period with expected improvement of contractility in the hours after PCI due to gradually reduced stunning.

The study will contribute to already existing information about the safety of levosimendan during acute myocardial infarction with special emphasis on cardiac arrythmias, hypotension and new ischaemic episodes.

The study will provide new information about the effect of levosimendan on inflammation which has been proposed to be an important part of the stunning mechanism.

The study will gain knowledge on the use of levosimendan in patients with cardiogenic shock. This is a group of patients which is almost always excluded from clinical trials and lack of information about pharmacological treatment is striking. We (see Appenix) and others (7) have used levosimendan in this setting as add-on therapy to catecholamines, but this should be done as a part of a placebo-controlled trial. Although the expected numer of patients in cardiogenic shock in the present trial is too low to provide information regarding mortality, the study will nevertheless give information about the effects of levosimendan on regional contractility, hemodynamics, potential side-effcts and the practical approach of combining levosimendan and catecholamines.

**Methods (see study protocol):**

Patients with acute myocardial infarction undergoing acute PCI will be screened for inclusion in the study. Patients developing symptoms and signs of left ventricular heart failure will be included in the study. Patients must have a reduced myocardial viability measured by echocardiography before inclusion in the study. Patients in cardiogenic shock will be assigned to a prospectively defined subgropup by stratified randomization. The study is a randomised double-blind study with parallel groups receiving a 24 hours infusion of levosimendan or placebo. This will be done by double-blind, double-dummy teqnique. The effect of the treatment will be validated by measurement of regional contractility (echocardiography), brain natriuretic peptide (BNP), symptom score and infarct size (isotope teqnique). The effect of levosimendan on inflammation markers will also be measured.

# Study center

Ullevål University Hospital, Oslo, Norway. Ullevål University Hospital is a high-volume coronary intervention center, staffed 24-hours a day, performing 600 acute PCIs per year. In addition, about 1500 routine PCIs are done yearly. The patients included in the study will be treated and monitored in either a coronary care unit or an intensive care unit.

## Study protocol

**Patient group**

Patient with acute myocardial infarction referred to Ullevål University hospital.

**Major inclusion criteria:** Patients who are hospitalised with acute ST-segment elevation myocardial infarction (STEMI: ECG definition) subjected to acute PCI

*or*

patients with non-ST segment elevation myocardial infarction subjected to PCI within 72 hours after start of chest pain.

*and all of the following (1-3)*

- - Revascularization by PCI with opening of an occluded coronary artery or balloon dilatation of a stenotic coronary artery presumed to be culprit lesion
  - Depressed contractility of the left ventricle (left ventricle ejection fraction < 40 %) assessed by echocardiography
  - Dyspnoea at rest at screening and *at least* one of the following signs of left ventricular failure (within 48 hours after PCI):
    - Pulmonary edema
    - Signs of marked pulmonary congestion on chest x-ray
    - Need for continuous-elevated positive airway-pressure ventilation (CPAP) or mechanical ventilation
    - Need for IV diuretics.
    - Oliguria (<0.5 ml/kg/hour) as a sign of hypoperfusion of the kidneys after volume therapy.

**Subgroup of patients with cardiogenic shock:**

A prospectively defined subgroup included by stratified randomisation.

**Inclusion criteria**

Additional inclusion criteria includes *both* of the following:

- - Systolic BP <90 mmHg after 60 min of adequate volume therapy or systolic BP between 90 and 100 mmHg with inotropic support by catecholamine infusion
  - Signs of hypoperfusion (low-output heart failure)
    - Oliguria (diuresis <0.5 ml/kg/hour)
    - Cold, clammy extremities
    - Reduced consciousness

**Exclusion criteria**

- Age below 20 years,
- Heart rate above 120 bpm
- Septic shock
- ARDS
- Creatinine >450 μmol/l
- Hepatic impairment
- Significant mechanical outlet obstruction
- Allergy against study drug medication or one of its ingredients
- Anaemia (Hb < 8 g/dl)
- Pregnancy

**Primary endpoints**

**Efficacy parameters**:

- - Changes from baseline to 5 days in Wall motion score-index measured by echocardiography.
  - Changes from baseline to 5 days in BNP (Brain natriuretic peptide).
  - Clinical composite at 5 days after the start of the study drug infusion, according to the physician’s global assessment measured as change from baseline to 5 days.

**Safety parameters**: Number of patients developing:

- - Hypotension: BP < 90 mm Hg or a drop in mean arterial BP > 10 mm Hg in patients with cardiogenic shock.
  - Tachycardia (heart rate above 120).
  - Atrial fibrillation.
  - Ventricular arrhythmia (VT, VF, TDP).
  - Ischemic episodes (ECG changes or chest pain demanding treatment).

# Secondary endpoints

# Efficacy parameters

- MACE: Time to death, non-fatal myocardial infarction or revascularization of the infarct related artery during the first 6 weeks and 6 month after start of the study drug infusion.
- Changes from baseline to 24 hours in Wall motion score-index measured by echocardiography
- Changes from baseline to 6 weeks in Wall motion score-index measured by echocardiography
- Changes from baseline to 24 hours in BNP
- Changes from baseline to 6 weeks in BNP
- Number of days in intensive or coronary care unit.
- Total number of days hospitalised.
- Time to re-hospitalization for decompensated heart failure.
- Clinical composite at 24 hours after the start of the study drug infusion, according to the physician’s global assessment measured as change from baseline to 24 hours.
- Infarct size measured at 6 weeks by gated spect (radioisotope technique).
- Changes of inflammation markers, haemostasis parameters and serum lactate from baseline to 24 hours, 5 days and 6 weeks.
- Changes of E/É ratio measured by echocardiography from baseline to 24 hours and 5 days.

**In a subgroup of patient *with* cardiogenic shock:**

- Improvement of hemodynamic parameters (CI, PCWP, and SVRI) from baseline to 24 and 48 hours.
- Number of days on intra-aortic-balloon counter pulsation treatment (IABP).
- Improvement of central venous oxygen saturation.
- Improvement of kidney function measured as creatinine clearance.
- Total mortality (number of patients alive at discharge).

**In patients *without* cardiogenic shock:**

- - The patient’s global assessment VAS-scale at 24 hours and at 5 days after the start of the study drug infusion.
  - Need for IV inotropes, at the clinicians decision, after start of the study drug infusion.

**Methods**

**Echocardiography**: Main endpoint in the study is improvement of regional contractility or wall motion score-index (WMSI). WMSI is measured by echocardiography including tissue Doppler imaging at baseline, 24 hours, 5 days and 6 weeks after the start of the study drug infusion.

**Blood analysis (see appendix II)**: A blood sample is frozen at baseline, after ending the infusion period, after 2 and 5 days and after 6 weeks after the start of the study drug infusion. BNP, haemostasis parameters and both pro- and anti-inflammation markers are analysed together at the end of the study in collaboration with Professor Harald Arnesen and Ingebjørg Seljeflot at Clinical Research Centre, Department of Heart-and Lung diseases, Ullevål University Hospital. Routine analyses like, troponin-T, lactate, creatinine, blood-gas analysis are done by the Department of Clinical Chemistry.

### Infarct size measurement

Infarct size will be assessed by technetium 99m-tetrofosmin myocardial SPECT (single photon emission computed tomography) imaging (8).

### Hemodynamic data

Hemodynamic data (cardiac index, cardiac power index, stroke volume, PCWP, SVRI, pulmonary artery pressure) and central venous oxygen saturation are collected by Swan-Ganz catheter. A Swan-Ganz catheter will only be used to monitor patients in cardiogenic shock.

The patient’s blood pressure will be monitored by non-invasive registrations except for patients in cardiogenic shock, which will be monitored, by invasive registrations.

**Assessment of symptoms and clinical findings:**

Global assessment is completed by the physician and the patient as change from baseline at 24 hours and at 5 days after the start of study drug treatment. A VAS-scale will be used to mark changes in symptoms. The physician will be asked to evaluate the degree of dyspnoea, respiratory frequencies, rales, third heart sound, hemodynamic assessment (cold/warm, clammy/dry extremities and urine output). Each patient will be scored on a scale from 1-4 describing the degree of symptoms. The patient will be evaluated again 24 hours after start of the study drug infusion. The patient is improved if the score is better and worsened if the score is worse. If the score is the same, the patient is unchanged. Worsening is defined as use of IV vasodilators, inotropic agents or IV diuretics. The patient will simply be asked to score symptoms on a VAS (1-10) scale at baseline, after 24 hours and 5 days.

**Registration of hospitalisation and MACE:**

Days in CCI/ICU and total length of stay in hospital will be measured. In-hospital mortality will be measured. Time to death, non-fatal myocardial infarction or revascularization is measured at 6 weeks and 6 month. The Norwegian death registry will be used to compare mortality at 6 month.

**6 weeks control:**

All patients will receive a 6 weeks follow-up consultation, which will include physical examination by a physician, echocardiography, blood analysis and gated spect (radio-isotope technique) analysis of infarct size.

**Safety variables**:

Safety variables (adverse events, HR, BP, ECG, lab, concomitant medication) are recorded during the study with special emphasize on blood pressure, cardiac arrhythmias, ischemic episodes an concomitant use of other inotropic drugs. This will be monitored during infusion of study drug and during the next 5 days of follow-up.

**Concomitant treatment:**

All patients will be treated with acute PCI and standard medical therapy according to national and international guidelines on treatment of acute ST-elevation myocardial infarction. The use of IV inotropes is restricted to the subgroup in cardiogenic shock. An exception is the use of noradrenalin as rescue therapy as described below. There are no further restrictions in the use of medical treatment. If the responsible clinician decides that indications for the use of IV inotropes is present, this will be noted in the case report form as a study endpoint. In patients in cardiogenic shock, all medical treatment is allowed in addition to the study drug. The use of IV inotropes in all patients will be collected in the case report form.

**Study medication:**

A bolus dose of 12 ug/kg for 60 minutes. The bolus dose is followed by a continuous infusion of 0,1 ug/kg/min for 24 hours. After 24 hours the infusion is turned off abruptly. Placebo is administered in the same way as levosimendan.

Noradrenalin must be mixed and ready for use before starting the study drug infusion. This is important because some patients receiving levosimendan develop vasodilatation and this is followed by a non-acceptable fall in blood pressure.

A non-acceptable fall in blood-pressure is defined as systolic BP below 80 mm Hg *and* a drop in mean arterial blood pressure > 10 mm Hg.

If the study drug is not well tolerated:

1. Decrease the infusion rate to 0,05 ug/kg/min. If this is not sufficient:
2. Start noradrenalin infusion (start dose: 0.03 μg/kg/min). If this is not sufficient:
3. Stop the infusion of the study drug.

**Total duration of study:**

The study drug infusion time is 25 hours. There are follow-up evaluations during the first 5 days in hospital and then at 6 weeks after the start of study drug treatment. The MACE end-point and total mortality will be recorded at 6 weeks and at 6 months. Frozen blood samples will be stored (biobanking) for 15 years (-70^0^C freezer) for potential further analyses.

**Number of patients:**

60 patients at Ullevål University hospital. A minimum of 14 patients in cardiogenic shock will be included. If necessary, the total number of patients in the study will be increased in order to reach the number of patients in cardiogenic shock.

**Statistics:**

The patients will be randomized and the data analyzed in collaboration with Professor Leif Sandvik, PhD, at Center of Statistics, Ullevål University hospital.

*Determination of Sample Size:* The total number of 60 patients in the study is based on the following: WMSI as a marker of regional contractility is the main endpoint of the study. This index is higher in patients with increasing number of damaged segments of the left ventricle. We have evaluated the method in patients with myocardial infarction including patients given levosimendan infusion post-PCI.

We postulate, from our preliminary experience with levosimendan in this clinical setting, a reduction of WMSI from 1.6 ± 0.2 (mean ± SD) to 1.4 ± 0.2 by adding levosimendan to standard therapy. This reduction represents a clinically important improvement of left-ventricular function. With α= 0.05 (two-sided test) and 1-β = 0.80, 30 patients in each group will be sufficient to detect a statistically significant difference between the groups.

*Randomization*: Patients meeting the entry criteria will be included in the study. A randomization code list will be computer generated by Professor Leif Sandvik, PhD, at Centre of Statistics. Patients in the predefined subgroup “cardiogenic shock” will be randomized to this subgroup by stratified randomization. The randomization code list will be kept in a safe at the Hospital Pharmacy. In a medical emergency situation requiring knowledge of the identity of the study medication given to a patient, the Hospital Pharmacist that will be available on a 24-hour basis will open the randomisation code.

*Populations for Analysis:*

The principal analysis will be performed on an intention-to-treat principle. The intention-to-treat population includes all patients who were randomized to the active treatment arm and the placebo treatment arm whether or not they received the treatment. The intention-to-treat population is the primary population for all baseline and efficacy analysis. The as-treatment population includes all patients who actually received any amount of study drug. The as-treated population is the primary patient population for the safety analyses.

**Adverse events**:

Any serious adverse event (SAE) in a patient administered the study drug will be classified as unlikely, possibly or probably related to the study drug. Both non-serious and serious AE will be collected during the study period on the case report form. Those events that are designated as study endpoints related to the underlying disease or secondary to invasive procedures resulting from disease progression and unrelated to the study drug medication, will be collected on the case report form. Any suspected unexpected serious adverse reaction (SUSAR) related to the study medication will be reported to the Norwegian Medicines Agency and to the EMEAs Eudra Vigilance Clinical Trial Module (EVCTM).

**Principle investigator:** Geir Øystein Andersen, MD, PhD.

**Steering committee:** Reidar Bjørnerheim (MD, PhD, Head of Department of Cardiology), Arild Mangschau (MD, PhD), Jan Eritsland (MD, PhD), Geir Øystein Andersen (MD, PhD) all at the Department of Cardiology and Alf Jonassen (MD, PhD) Section of Cardiothoracic Anaesthesia, Department of Anaesthesia, Ullevål University hospital.

**Safety committee**: Any serious adverse events (SAE) will be reported to the safety committee, which will decide whether the SAE is regarded as a SUSAR or not. Members of the committee are: Professor Dan Atar and Kjetil Steine (MD, PhD), Aker University hospital.

**Country** Norway

**Draft time plan**

2004-06: Approval from the Regional ethics committee, The Norwegian Data Inspectorate and The Norwegian Medicines Agency.

June 2006: Start of inclusion.

Des 2009: End of inclusion.

2010: Publications of the results.

**References:**

1. Kivikko M, Lehtonen L, Colucci WS. Sustained hemodynamic effects of intravenous levosimendan. Circulation 2003; 107: 81-6.
2. Follath F, Cleland JG, Just H, Papp JG, Scholz H, Peuhkurinen K, et al. Efficacy and safety of intravenous levosimendan compared with dobutamine in severe low-output heart failure (the LIDO study): a randomised double-blind trial. Lancet 2002; 360: 196-202.
3. Moiseyev VS, Poder P, Andrejevs N, Ruda MY, Golikov AP, Lazebnik LB, et al. Safety and efficacy of a novel calcium sensitizer, levosimendan, in patients with left ventricular failure due to an acute myocardial infarction. A randomized, placebo-controlled, double blind study (RUSSLAN). Eur Heart J 2002; 23: 1422-32.
4. Bolli R. why myocardial stunning is clinically important. Basic Res Cardiol 1998; 93: 169-72.
5. Nieminen MS, Akkila J, Hasenfuss G, Kleber FX, Lehtonen LA, Mitrovic V, et al. Hemodynamic and neurohumoral effects of continuous infusion of levosimendan in patients with congestive heart failure. J Am Coll Cardiol 2000; 36: 1903-12.
6. O'Connor CM, Gattis WA, Uretsky BF, Adams KF, Jr., McNulty SE, Grossman SH, et al. Continuous intravenous dobutamine is associated with an increased risk of death in patients with advanced heart failure: insights from the Flolan International Randomized Survival Trial (FIRST). Am Heart J 1999; 138: 78-86.
7. Delle Karth G, Buberl A, Geppert A, Neunteufl T, Huelsmann M, Kopp C, et al. Hemodynamic effects of a continuous infusion of levosimendan in critically ill patients with cardiogenic shock requiring catecholamines. Acta Anaesthesiol Scand 2003; 47: 1251-6.
8. Halvorsen S, Mülller C, Bendz B, Eritsland J, Brekke M and Mangschau A. Left ventricular function and infarct size 20 months after primary angioplasty for acute myocardial infarction. Scand Cardiovasc J. 2001; 35: 379-84.
