# Supplementary material for: Association of Interleukin 8 and Myocardial Recovery in Patients with ST-Elevation Myocardial Infarction Complicated by Acute Heart Failure
Source: PLoS One. 2014 Nov 12;9(11):e112359. doi: 10.1371/journal.pone.0112359 (PMC4229310; doi:10.1371/journal.pone.0112359)
Supplement: Protocol S2 — LEAF Studyprotocol First Amendment S2. (DOC) [file pone.0112359.s003.doc]

# Amendment to CLINICAL STUDY PROTOCOL

Title: Safety and efficacy of levosimendan in patients with acute myocardial infarction complicated by symptomatic left ventricular failure (The LEAF study).

Sponsors protocol nr: 0105

Date: 2006-11-20

## From 2006-11-20 the inclusion criteria of the LEAF study have been changed.

## Inclusion in the study is based on three main objectives.

## Acute PCI due to an acute myocardial infarction.

## Depressed contractility of the left ventricle (left ventricle ejection fraction < 40 %) assessed by echocardiography.

## Symptoms and signs of heart failure.

##

## In the revised protocol criteria number two has been changed. In the original protocol the left ventricle ejection fraction (LVEF) had to be less than 40 % assessed by echocardiography. This has been changed in the revised protocol to the following: Regional left ventricular contractility must be assessed by segmental analysis. The left ventricle is divided in to 16 segments. Signs of decreased wall-motion (hypokinesia, akinesia or dyskinesia) must be present in at least 3 of 16 segments evaluated by echocardiography.

##

## Rationale for the revision of the protocol:

## We have so far included 4 of 60 patients planned to be included in the study. We have screened about 10 patients for the study with clinical symptoms of heart failure after PCI treatment of acute myocardial infarction. Because of the present inclusion criteria (Nr. 2) we had to exclude patients with large myocardial infarction due to occlusion of the left anterior descendent artery (LAD) because the rest of the myocardium compensated to such an extent that the left ventricle ejection fraction (LVEF) was estimated to be above 40 %. Approximately 50% of patients hospitalized for acute decompensated heart failure have preserved systolic function (measured as LVEF <40%) according to the large ADHERE registry of acute heart failure had an LVEF above 40 % (1). Patients with previously normal heart can have a substantial decrease in myocardial viability in the early phase of acute myocardial infarction and still have an LVEF > 40 %. Ejection fraction is probably a more suitable inclusion criteria in studies of chronic heart failure when the myocardial remodelling and stunning period are over. A segmental analysis (16 segments) with assessment of regional viability is used in the LEAF study to evaluate the main end-point which is wall-motion score index. This method of evaluating myocardial function has been evaluated in large heart failure studies (2). The revised protocol will include analysis of segments as performed in the newly published Norwegian ASTAMI study (3) also as *inclusion criteria* instead of LVEF. The revision of the protocol has been done in order to be able to include symptomatic patients in the early phase of an acute myocardial infarction with signs of decreased myocardial viability whether or not the LVEF is below or above 40 %.

The old and the revised inclusion criteria are summarised below.

**Original protocol:**

| **Patient group**  **Major inclusion criteria:** | Patients who are hospitalised with acute ST-segment elevation myocardial infarction subject to acute PCI  *or*  patients with non-ST segment elevation myocardial infarction subject to PCI within 72 hours after start of chest pain.  *and all of the following (1-3)*   - - Revascularization by PCI with opening of an occluded coronary artery or balloon dilatation of a stenotic coronary artery presumed to be culprit lesion.   - **LVEF must be less than 40% measured by echocardiography.**   - Dyspnoea at rest at screening and *at least* one of the following signs of left ventricular failure (within 48 hours after PCI):     - Pulmonary edema     - Signs of marked pulmonary congestion on chest x-ray     - Need for CPAP or mechanical ventilation     - Need for IV diuretics.     - Oliguria (<0.5 ml/kg/hour) as a sign of hypoperfusion of the kidneys (after volume therapy) |
| --- | --- |

## Revised protocol:

| **Patient group**  **Major inclusion criteria:** | Patients who are hospitalised with acute ST-segment elevation myocardial infarction subject to acute PCI  *or*  patients with non-ST segment elevation myocardial infarction subject to PCI within 72 hours after start of chest pain.  *and all of the following (1-3)*   - - Revascularization by PCI with opening of an occluded coronary artery or balloon dilatation of a stenotic coronary artery presumed to be culprit lesion.   - **Signs of decreased wall-motion (hypokinesia, akinesia or dyskinesia) must be present in at least 3 of 16 segments.**   - Dyspnoea at rest at screening and *at least* one of the following signs of left ventricular failure (within 48 hours after PCI):     - Pulmonary edema     - Signs of marked pulmonary congestion on chest x-ray     - Need for CPAP or mechanical ventilation     - Need for IV diuretics.     - Oliguria (<0.5 ml/kg/hour) as a sign of hypoperfusion of the kidneys (after volume therapy) |
| --- | --- |

1. Yancy C et al. Clinical Presentation, Management, and In-Hospital Outcomes of Patients Admitted With Acute Decompensated Heart Failure With Preserved Systolic Function. JACC 2006;47:76
2. Gislason GH et al. The reliability of echocardiographic left ventricular wall motion index to identify high-risk patients for multicenter studies. Echocardiography 2006; 23: 1.
3. Lunde K et al. Intracoronary injection of mononuclear bone marrow cells in acute myocardial infarction. NEJM 2006;355:1195.
