# Supplementary material for: Association of Interleukin 8 and Myocardial Recovery in Patients with ST-Elevation Myocardial Infarction Complicated by Acute Heart Failure
Source: PLoS One. 2014 Nov 12;9(11):e112359. doi: 10.1371/journal.pone.0112359 (PMC4229310; doi:10.1371/journal.pone.0112359)
Supplement: Protocol S3 — LEAF Studyprotocol Second Amendment S3. (DOC) [file pone.0112359.s004.doc]

# Amendment to CLINICAL STUDY PROTOCOL

Title: Safety and efficacy of levosimendan in patients with acute myocardial infarction complicated by symptomatic left ventricular failure (The LEAF study).

Sponsors protocol nr: 0105

Date: 2008-01-30

## Amendment to the study protocol: Secondary endpoints

# *The original protocol:*

**Secondary endpoints:**

In a subgroup of patient *with* cardiogenic shock:

- Improvement of hemodynamic parameters (CI, PCWP, and SVRI) from baseline to 24 and 48 hours.

**Methods**: Hemodynamic data (Cardiac index, stroke volume, PCWP, SVRI etc.) and central venous oxygen saturation are collected by a pulmonary artery-catheter (Swan-Ganz catheter) in a subgroup of patients in cardiogenic shock.

# *Revision of the protocol:*

**Secondary endpoints:**

In a subgroup of patient *with* cardiogenic shock:

- When available from a pulmonary artery-catheter: Improvement of hemodynamic parameters (CI, PCWP, and SVRI) from baseline to 24 and 48 hours

**Methods**: In a subgroup of patients in cardiogenic shock, hemodynamic data (Cardiac index, stroke volume, PCWP, SVRI etc.) and central venous oxygen saturation are collected by a pulmonary artery-catheter (Swan-Ganz catheter) when available. If a pulmonary artery-catheter is either not tolerated by the patients (e.g. due to arrhythmias) or for practical reasons not available, the patients should be included in the study and monitored by echocardiography and repeated samples form a central venous line.

## Rationale for the revision of the protocol:

We have so far included 20 patients in the study, but only 3 patients in cardiogenic shock. Two patients in cardiogenic shock were screened, but excluded from the study because insertion of a pulmonary artery-catheter was complicated by repeated ventricular arrhythmias, which required emergency treatment. These patients are extremely vulnerable to arrhythmias and in order to avoid exclusion of these patients the protocol has been revised accordingly. In the revised protocol, pulmonary artery-catheter monitoring is no longer mandatory in patients in cardiogenic shock, but should be inserted in as many patients as possible.

It is emphasised that this amendment does not change the inclusion criteria of patients in cardiogenic shock, which are based on clinical examination and not invasive measurements.

This amendment was recommended by the Steering committee on January 22, 2008 and approved by the Safety committee on January 23, 2008.
